# Diurnal liver mass is associated with ribosome biogenesis

**DOI:** 10.18632/oncotarget.22255

**Published:** 2017-11-01

**Authors:** Flore Sinturel, Frédéric Gachon

**Affiliations:** Frédéric Gachon: Department of Diabetes and Circadian Rhythms, Nestlé Institute of Health Sciences, Lausanne, Switzerland; Faculty of Life Sciences, Ecole Polytechnique Fédérale de Lausanne, Lausanne, Switzerland

**Keywords:** circadian rhythms, feeding rhythms, liver, ribosome biogenesis, ribosomal RNA

Because of the rotation of the Earth around its own axis, most of the leaving species on our planet are subjected to day-night cycles with a period of 24 hours. As a consequence, nearly all aspects of their behavior and metabolism are coordinated in a rhythmic fashion with a period of about a day. This rhythmicity is coordinated by a network of interconnected self-sustained cell-autonomous oscillators called the circadian clock. At the level of the whole organism, the circadian system is organized in a hierarchical manner with a master clock within the suprachiasmatic nuclei (SCN) of the hypothalamus. These SCN are receiving light input via the retina and transmitting timing signals to secondary oscillators in peripheral tissues, thus ensuring phase coherence within the body. In turn, these peripheral clocks coordinated key metabolic functions in each respective organ. As a consequence, perturbation of circadian rhythms by experimental or social conditions is associated with diverse pathologies including obesity and type 2 diabetes. In addition to light-dark cycles, feeding rhythm is an important synchronizer of peripheral clocks and can even override the effect of the SCN in synchronizing peripheral tissues [1]. This indicates that coordinating the timing of metabolism is a major purpose of peripheral clocks.

The liver clock is one of the most robust circadian peripheral organ in mammals, probably because of its crucial role in the diurnal processing and storage of nutrients. A daily change in liver mass has been observed in different species, from birds to human. The characterization of this entire liver oscillation in mouse revealed that it is accompanied with daily changes in hepatocytes size and required the alignment of the feeding cycles with the circadian core clock [2], likely through rhythmic circulating factors that activates the remodeling of the liver cytoskeleton [3]. In addition, this diurnal variation in liver size disappeared under fasting conditions and has been therefore associated with feeding rhythm [4]. However, the liver macromolecules contributing to liver mass changes were not yet characterized. Among the macromolecules showing a diurnal patterns that disappears under unsynchronized circadian and feeding rhythms induced by day-time feeding condition, RNA and protein appeared the ones showing the more important fluctuation [2]. As protein constitutes the majority of liver dry mass, oscillation in cellular protein accumulation is likely associated with liver size changes. Indeed, protein production in mouse hepatocytes rose sharply at night, followed by equivalent protein degradation during the day [2]. Protein synthesis is performed through the translation of mRNA by ribosomes, a complex assembly of ribosomal proteins (RP) and ribosomal RNA (rRNA). Ribosome biogenesis is a complex and highly controlled process and the efficient production of functional ribosomal subunits requires the coordination of rRNA and RP synthesis. Interestingly, both RP and rRNA fluctuate in mouse liver, through rhythmic synthesis or polyadenylation-dependent degradation, respectively [2]. Indeed, while extensive literature described the role of circadian and feeding rhythms on rhythmic transcription, recent evidences also point out to a rhythmic orchestration of mRNA translation and ribosome biogenesis [5]. This rhythmic RP synthesis and assembly is controlled by the Target of Rapamycin pathway (TOR) which is activated by nutrient cues, mainly amino acids. As a consequence, the TOR pathway is rhythmically activated, with a rhythm dependent on feeding rhythms [2] and only slightly impacted by circadian clock disruption [6]. It therefore appeared that feeding rhythms plays a key role in liver size regulation. However, it remains to decipher the benefit of the liver mass oscillation for the metabolism. The liver needs a cyclic replacement of its components, *i.e.* regulatory proteins and enzymes participating to the nutrients processing and detoxification of harmful substances, to deal with the feeding rhythms imposed by behavior and sleeping rhythms. In this context, the oscillating liver mass could participate to a precise “maintenance program” and give the ability to the liver to adapt to the nutrition state. For example, glucose tolerance is dependent on the time of day, glucose being in mouse more tolerated during the beginning of the night and less in the beginning of the day [7]. This phenomenon could explained glucose intolerance in shift workers, associated to their higher prevalence for obesity and type 2 diabetes [8].
